# Small duct autoimmune sclerosing cholangitis and Crohn colitis in a 10-year-old child. A case report and review of the literature

**DOI:** 10.1186/1746-1596-7-100

**Published:** 2012-08-14

**Authors:** Erling Peter Larsen, Allan Bayat, Mogens Vyberg

**Affiliations:** 1 Department of Pediatrics, Aalborg hospital, Aarhus University Hospital, Aalborg, Denmark; 2 Institute of Pathology, Aalborg hospital, Aarhus University Hospital, P.O.Box 561, Aalborg, DK-9100, Denmark

**Keywords:** Autoimmune sclerosing cholangitis, Crohn colitis, Granulocytic epithelial lesion, Overlap syndrome

## Abstract

**Abstract:**

Autoimmune sclerosing cholangitis is an overlap syndrome characterized by features of both autoimmune hepatitis and primary sclerosing cholangitis, the latter usually involving the large bile ducts. Autoimmune sclerosing cholangitis occurs more often in children than in adults and is frequently associated with inflammatory bowel disease, predominantly ulcerative colitis. We report a unique case of a 10-year-old Danish boy with severe small duct autoimmune sclerosing cholangitis and synchronic Crohn colitis. He was referred with a history of weight loss, abdominal pain, vomiting and diarrhea. Biochemical anomalies included elevated alanine aminotransferase, γ-glutamyl transferase and immunoglobulin G levels and the presence of smooth muscle antibodies and perinuclear antineutrophil cytoplasmic antibodies but normal alkaline phosphatase. Liver biopsy specimen revealed features of both autoimmune hepatitis and sclerosing cholangitis, the latter characterized by acute, hyperplastic and destructive inflammation – granulocytic epithelial lesion – of the small ducts. Magnetic resonance cholangiography was normal. Colonoscopic biopsies showed chronic inflammatory changes of the caecum and the ascending and transverse colon compatible with Crohn disease. Ursodeoxycholic acid and immunosuppressive treatment was initiated and within four weeks of treatment the general condition improved. Normalization of aminotransferase was seen at 21 weeks and γ-glutamyl transferase at 72 weeks after first admittance, while immunoglobulin G remained slightly increased.

**Virtual slides:**

The virtual slide(s) for this article can be found here: http://www.diagnosticpathology.diagnomx.eu/vs/1418596609736470

## Background

Immune-mediated liver diseases fall into two broad categories, those with a hepatitic predominance: autoimmune hepatitis (AIH), and those with a predominance of cholestatic features: primary biliary cirrhosis (PBC) and primary sclerosing cholangitis (PSC). AIH is characterized by elevated serum aminotransferases, hypergammaglobulinaemia (primarily immunoglobulin G), circulating autoantibodies (primarily antinuclear antibodies (ANA) and smooth muscle cell antibodies (SMA)) and interface hepatitis on liver biopsy specimen, while markedly elevated serum alkaline phosphatase (ALP), circulating antimitochondrial antibodies (AMA), or biliary changes on liver biopsy specimen are normally not present [1]. In contrast, PBC and PSC are characterized by cholestatic biochemistry, occurrence of AMA (PBC only), and histological biliary changes such as granulomatous cholangitis (PBC only) or fibroobliterative cholangitis (mainly PSC), leading to ductopenia and biliary cirrhosis. However, PBC and PSC may also reveal features of AIH, which are sufficiently pronounced to qualify for so-called overlap syndromes, even though there are no established definitions for these [2]. A scoring system established by the International Autoimmune Hepatitis Group (IAIHG) for research purposes [1] has been widely used in the clinical practise to classify patients as having “definite AIH”, “probable AIH” or “not AIH”. The scoring system is, however, not directly applicable for overlap syndromes.

The AIH/PSC overlap syndrome, also designated autoimmune sclerosing cholangitis (AISC) is occasionally seen in adults [2-4], but more often occur in children, where up to about 50% of patients presenting with AIH reveal radiological features of cholangiopathy [5]. Even among AIH patients with normal cholangiogram, histological biliary changes have been found in about 30% [5]. In other studies, cases have been classified as AISC even though the cholangiopathy was limited to the small ducts [6-8].

Both PSC and AISC frequently occur in combination with inflammatory bowel disease (IBD) [2], typically ulcerative colitis (UC) while fewer cases of indeterminate colitis (IC) and Crohn disease (CD) has been described [5,7,9-11]. We present a case of a 10-year-old boy, diagnosed as AISC with an unusual, severe small duct lesion and concomitant Crohn colitis.

## Case presentation

The patient, who had previously been in good health, was admitted to the pediatric department because of weight loss, upper abdominal pain, vomiting and diarrhea during 4 months. There was a family history of immune-mediated disorders: The father of the patient had been diagnosed with sarcoidosis and the father’s brother had CD.

The initial physical examination revealed an alert but tired and pale boy. Weight was 39 kg and height 146 cm. Pulse rate and blood pressure were normal. There was no hepatosplenomegaly or jaundice.

**Figure 1:**
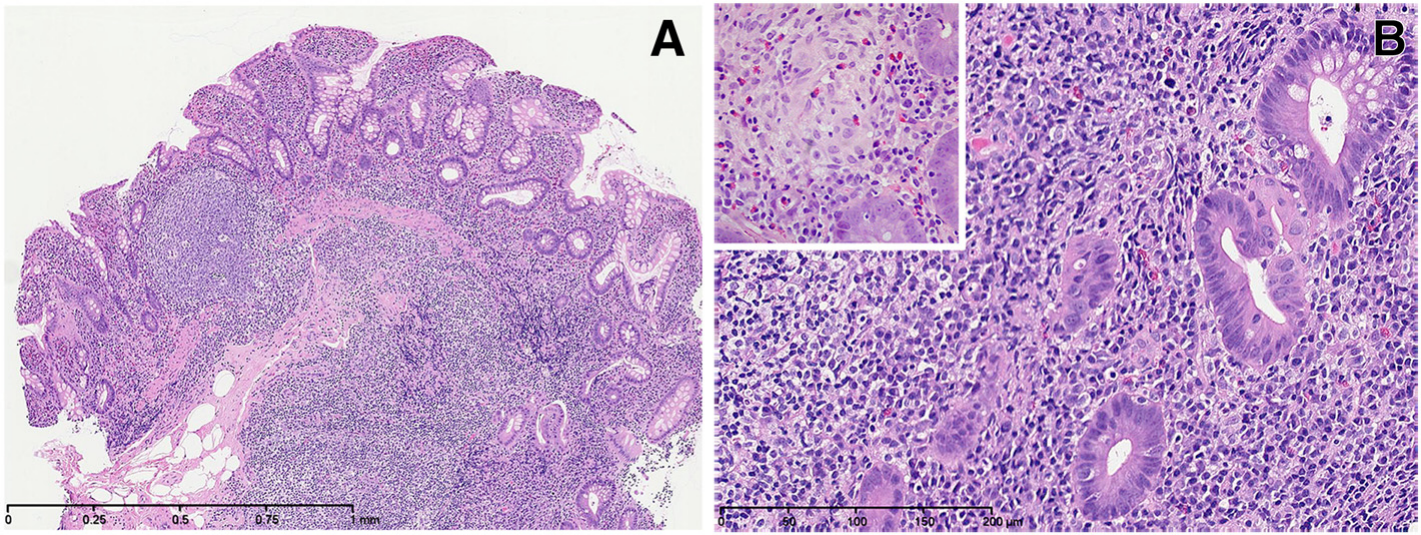
**Endoscopic biopsy specimen from right colon showing changes compatible with Crohn disease. A**. Colon mucosa with crypt atrophy and irregularity, and lymphoid hyperplasia (H&E, bar = 1 mm). **B**. Higher magnification revealing crypt destruction and in the lamina propria severe accumulation of lymphocytes and plasma cells as well as a single small epithelioid cell granuloma (inset) (H&E, bar = 200 μm).

**Figure 2:**
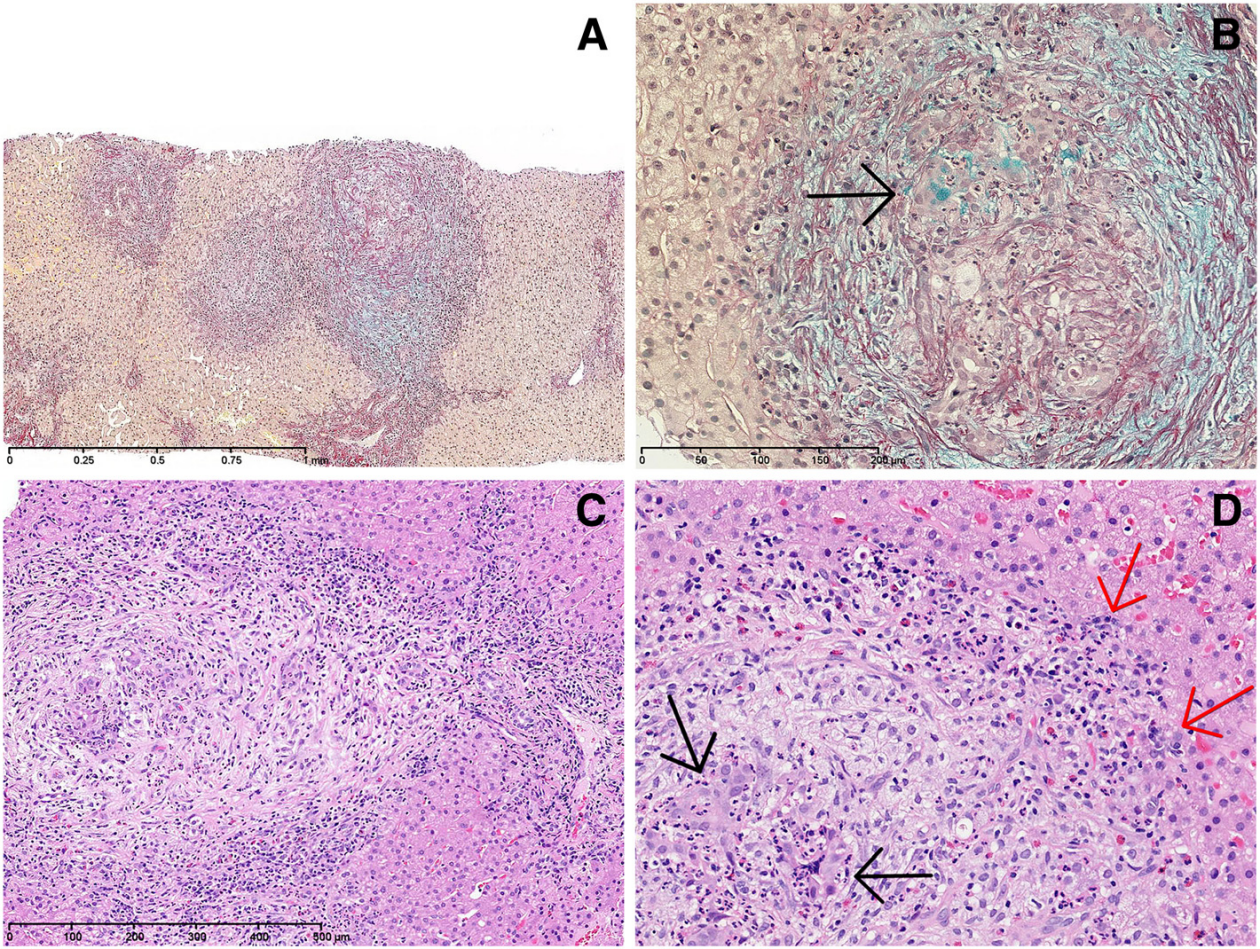
**Needle biopsy specimen from liver with changes compatible with autoimmune sclerosing cholangitis. A**. Marked portal enlargement due to inflammation, edema and portal/periportal fibrosis (Alcian-Picro-Sirius, bar = 1 mm). **B**. In the portal/periportal fields ductular reaction with severe epithelial hyperplasia, loss of polarity and focal cytoplasmic mucin accumulation (arrow) is seen (Alcian-Picro-Sirius, bar = 200 μm). **C**. The most pronounced inflammation occurs in small portal tracts around which also a severe lymfo-plasmacytic interface inflammation is seen (H&E, bar = 500 μm). **D**. Focally, bile duct epithelium is necrotic and infiltrated with neutrophils (black arrows). Pronounced interface hepatitis is present (red arrows) (H&E).

At the admission, the erythrocyte sedimentation rate (ESR) was 75 mm/hr (normal 2–13); serum alanine aminotransferase (ALAT) 75 U/L (normal 5–35); γ-glutamyl transferase (GGT) 239 U/L (normal 10–45); immunoglobulin G (IgG) 48.0 g/L (normal 6.08-15.72); SMA 1:320; ANA negative; serum perinuclear antineutrophil cytoplasmic antibodies (pANCA) 1:80; cytoplasmic (c) ANCA negative; fecal calprotectin 1268 mg/kg (normal 0–50); ALP, bilirubin, albumin and blood platelets were normal; tests for viral hepatitis and bacterial growth in stools were negative; test for liver-kidney-microsomal antigen-1 was not available. Based on the scoring system proposed by the International Autoimmune Hepatitis Group (IAIHG) [1] the patient fulfilled the criteria for the diagnosis of “definite” AIH (aggregate score = 21; however, see discussion). Magnetic resonance cholangiopancreatography (MRCP) showed no cholangiographic abnormalities. Endoscopic retrograde cholangiopancreatography (ERCP) was not carried out. Colonoscopy showed scattered reddened mucosa with small aphtoid ulcers in the caecum and the ascending and transverse colon. Biopsy specimens from these areas revealed active chronic inflammation compatible with CD (Figure1). A liver needle biopsy specimen revealed features of both AIH and PSC (Figure2): Disturbed architecture with marked enlargement of mainly small portal tracts due to inflammation, edema and portal/periportal fibrosis. The inflammatory infiltrates consisted of a mixture of lymphocytes, plasma cells, histiocytes, and eosinophilic and neutrophilic granulocytes. Granulomas were not found. In several fields ductular reaction with severe epithelial hyperplasia, loss of polarity and focal cytoplasmic mucin accumulation was seen. Some epithelial structures appearing like reactive bile ductules were embedded in a myxoid fibrotic tissue. Immunohistochemical analysis revealed strong expression of keratin 19 and focal expression of MUC5AC (but not MUC2) in the reactive ductules. The epithelium of some interlobular bile ducts was focally necrotic with neutrophil infiltration, but bile duct loss could not be demonstrated. Around these portal tracts a severe interface inflammation was seen with hydropic and rosetting juxtaportal hepatocytes surrounded by a lymphoplasmacytic infiltrate. Other portal tracts, particularly the larger ones, only showed minor inflammation, no bile duct changes, and no interface inflammation. Bridging necrosis or fibrosis was not seen. The parenchyma in zones I and II showed only slight reactive changes. Copper binding protein and Mallory-Denk bodies were not found. Immunohistochemical analysis showed 1–3 IgG4 positive plasma cells in the inflamed portal tracts. Treatment with prednisolone 1 mg/kg daily and ursodeoxycholic acid (UDCA) 10 mg/kg daily was started. Within four weeks of treatment the patient’s general condition improved and he resumed school activities. After ten weeks of treatment ESR, ALAT and IgG still indicated disease activity (Table1). The patient started treatment with azathioprine 1.5 mg/ kg daily while the dosage of prednisolone was slowly reduced to 7.5 mg every second day. This resulted in a normalization of ALAT and ESR while IgG was still slightly elevated around 18 g/L. The dosage of azathioprine was then raised to 2 mg/kg daily. At the latest examination 72 weeks after diagnosis, the general condition was good but IgG remained slightly increased. A slight increase in ALAT and ESR was ascribed to an acute viral infection. In order to rule out progressive biliary damage an MRCP was done, which was still normal.

**Table 1:** Laboratory investigations in the patient at the time of diagnosis and after 2–72 weeks of treatment

**Laboratory**	**Normal**	**Units**	**Values at time**	**Values during treatment (weeks)**					
**Investigation**	**values**		**of diagnosis**	**2**	**6**	**8**	**12**	**21**	**72**
ESR	2-13	mm/hr	75	63	50	46	30	13	28
ALAT	5-35	U/L	106	490	170	129	42	27	43
GGT	10-45	U/L	262	239	NA	NA	NA	NA	17
IgG	6.08-15.72	g/L	48.0	29.1	25.5	21.2	19.4	17.9	17.3

ALAT: Alanine aminotransferase; ESR: Erythocyte sedimentation rate; GGT: γ-glutamyl transferase; NA: not available.

## Discussion

The typical features of PSC (with or without concomitant AIH) are intrahepatic and/or extrahepatic large duct involvement with cholangiographically characteristic multifocal strictures and segmental dilatation or beading. Alternatively, PSC may present as histologically characteristic bile duct changes similar to long-standing large duct obstruction and eventually ductopenia, but with negative cholangiogram, thus designated small duct PSC [5,12,13]. Among PSC patients with positive cholangiogram, an overlap with definite or probable AIH has in adults (or mainly adults) been diagnosed in 1.4%-35% [4,6,14-16] while in children diagnosed in 28%-49% of the cases [5,7,9,17,18]. However, PSC in children is frequently characterized serologically by florid AIH-like features including ANA and SMA, elevated IgG and histologically by interface hepatitis, wherefore these children may be diagnosed as AIH in the absence of cholangiographic studies or with an initially normal cholangiogram, unless characteristic bile duct changes are identified histologically. Moreover, despite bile duct involvement, cholestatic biochemistry in children is relatively rare, many having normal ALP and in some cases even normal GGT at presentation [9]. Hence, when either AIH or PSC is diagnosed in children, it may take years before an overlap syndrome, i.e. AISC, is diagnosed [16]. To further confuse the picture, “incidental” histologic biliary changes may occur in classic AIH, which does not appear to develop features of PSC. Czaja and Carpenter [19] presented 84 AIH patients (including a small but unspecified number of children) among which ten appeared histologically to have destructive cholangitis or ductopenia. However, the AIH treatment response was not dependent on bile duct injury wherefore the authors were reluctant to consider the lesions as PSC. They proposed that the bile duct lesions were coincidental findings associated with classic disease, or weak expressions of a variant syndrome. In the study of Gregorio et al. [5] 8/26 children (31%) with AIH (in the absence of radiologic features of cholangiopathy) showed histologic biliary features of which one was classified as (small duct) PSC.

The diagnostic criteria formulated by the IAIHG [1] do not readily allow for a distinction between AIH (i.e., without PSC) and AISC, and is not directly applicable in children. Thus, the score for our patient could be calculated to 21, based on – among others – an ALP:ALAT ratio <1.5, but ALP is often normal in children with PSC and should probably be substituted with GGT [9,20].

In the current case, the liver changes are those of a chronic cholangiopathy within the spectrum of PSC. Peculiar to our patient was the severe inflammatory changes in relation to the small intrahepatic bile ducts with heavy neutrophil infiltration and features of destructive injury and marked hyperplasia, resembling the granulocytic epithelial lesion (GEL) in autoimmune pancreatitis (AIP) type 2 [21], a feature which we have previously seen published only once, in a case study of Grammatikopoulos et al. [22], who described a 13-year-old boy with concomitant Crohn colitis and AISC with GEL, who showed long-term remission of liver disease after steroid treatment.

Bile duct injuries in PSC and AISC may be considered multifactorial, potentially involving immune-mediated, chemical, genetic, ischaemic, and infectious mechanisms, as reviewed by Krones et al. [23]. In the above mentioned case [22] as well as ours, the GELs and the remission after steroid treatment supports an immune-mediated disease in line with AIP type 2, which interestingly is associated with IBD [8]. This is in contrast to the IgG4-associated PSC and hepatic inflammatory pseudotumour, which may occur concomitant with AIP type 1 [24].

The hyperplastic bile duct lesions described in this case bear some resemblance to the so-called hepatitis-associated bile duct lesion type 3 [25], which in fact appears to be an interface hepatitis related liver-cell lesion. However, in the current biopsy specimen the cytoplasmic cytokeratin 19 as well as mucin and MUC5AC expression in some of the lesions indicates that the cells are more likely of bile ductular origin. Normal small bile ducts and ductules do not secrete mucin or express mucin core proteins (MUCs). Overexpression in the intrahepatic biliary tree and cultured biliary epithelial cells has been shown to be due to e.g., bacterial infection, presumably via lipopolysaccharide induced synthesis of tumour necrosis factor-α and activation of protein kinase C [26].

Among children with AISC, almost half have concomitant IBD, but CD is relatively rarely specified. In the study of Gregorio et al. [5] one child with AIH and probable small duct PSC associated with CD is mentioned. Feldstein et al. [9] described that out of 40 children with PSC, 14 had overlap with AIH, and 8 out of 52 had CD, but did not specify how many had concomitant AISC and CD. Miloh et al.[7] described 12 children with AISC of which 6 had concomitant IBD but it is not clear how many had CD. In none of this cases were GEL like changes described.

No guidelines for the treatment of AISC (isolated or in combination with IBD) have been established, but a combination of UDCA and immunosuppressive treatment has been recommended [27]. The present trend is to target the treatment to one of the diseases and then to adjust it depending on symptoms and side effects [28]. Patients with large duct AISC have significantly worse prognosis than patients with AIH or large duct PSC [2,5,9]. Small duct PSC show a more protracted course than large duct PSC [7,12], but the number of patients with small duct AISC is too small for a prognostic evaluation. However, one may speculate that the AIP type 2-like liver damage signifies a better response to steroids compared to PSC in general.

## Conclusion

This report describes a rare case of AISC with concomitant Crohn colitis in a child with a unique hyperplastic and destructive cholangitis, resembling GEL in AIP type 2, limited to the small intrahepatic bile ducts.

## Consent

Written informed consent was obtained from the patient and his parents for publication of this Case Report and any accompanying images. A copy of the written consent is available for review by the Editor-in-Chief of this journal.

## Abbreviations

AIH: Autoimmune hepatitis; AISC: Autoimmune sclerosing cholangitis; ALAT: Alanine aminotransferase; ALP: Alkaline phosphatase; ANA: Antinuclear antibody; ANCA: Antineutrophil cytoplasmic antibody; CD: Crohn disease; ERCP: Endoscopic retrograde cholangiopancreatography; ESR: Erythrocyte sedimentation rate; GGT: γ-glutamyl transferase; IAIHG: International Autoimmune Hepatitis Group; IBD: Inflammatory bowel disease; IC: Indeterminate colitis; IgG: Immunglobulin G; INR: International normalized prothrombin ratio; LKM1: Anti-liver kidney microsomal type 1 antibody; MRCP: Magnetic resonance cholangiopancreatography; NA: Not available; PBC: Primary biliary cirrhosis; PSC: Primary sclerosing cholangitis; SMA: Smooth muscle cell antibody; UC: Ulcerative colitis; UDCA: Ursodeoxycholic acid.

## Competing interests

The authors declare that they have no competing interests.

## Authors’ contributions

EPL, AB and MV equally participated in the conception of the idea and writing of the manuscript. MV performed the histopathological interpretation of the liver- and colon biopsies. All authors have read and approved the final manuscript.
